# Association of HIV Intervention Uptake With HIV Prevalence in Adolescent Girls and Young Women in South Africa

**DOI:** 10.1001/jamanetworkopen.2022.8640

**Published:** 2022-04-22

**Authors:** Kaymarlin Govender, Sean Beckett, Tarylee Reddy, Richard G. Cowden, Cherie Cawood, David Khanyile, Ayesha B. M. Kharsany, Gavin George, Adrian Puren

**Affiliations:** 1 Health Economics and HIV and AIDS Research Division, University of KwaZulu-Natal, Durban, South Africa; 2 Biostatistics Research Unit, South African Medical Research Council, Durban, South Africa; 3 Human Flourishing Program, Harvard University, Cambridge, Massachusetts; 4 Epicentre AIDS Risk Management Limited, Cape Town, South Africa; 5 Centre for the AIDS Programme of Research in South Africa, University of KwaZulu-Natal, Durban, South Africa; 6 National Institute for Communicable Diseases, Johannesburg, South Africa; 7 National Priority Programmes, National Health Laboratory Services, Johannesburg, South Africa

## Abstract

**Importance:**

In South Africa, adolescent girls and young women aged 15 to 24 years are among the most high-risk groups for acquiring HIV. Progress in reducing HIV incidence in this population has been slow.

**Objective:**

To describe HIV prevalence and HIV risk behaviors among a sample of adolescent girls and young women and to model the association between exposure to multiple or layered interventions and key HIV biological and behavioral outcomes.

**Design, Setting, and Participants:**

This cross-sectional survey was conducted between March 13, 2017, and June 22, 2018, in 2 districts in Gauteng province and in 2 districts in KwaZulu-Natal province in South Africa. A stratified cluster random sampling method was used. Participants included adolescent girls and young women aged 12 to 24 years who lived in each sampled household. Overall, 10 384 participants were enrolled in Gauteng province and 7912 in KwaZulu-Natal province. One parent or caregiver was interviewed in each household. Data analysis was performed from March 12, 2021, to March 1, 2022.

**Exposures:**

DREAMS (Determined, Resilient, Empowered, AIDS-free, Mentored, and Safe)-like interventions.

**Main Outcomes and Measures:**

The primary outcome was HIV prevalence. HIV status was obtained from laboratory-based testing of peripheral blood samples. Secondary outcomes included HIV testing and antiretroviral therapy uptake as well as numerous HIV risk variables that the DREAMS program sought to improve, such as pregnancy, sexually transmitted infection, intimate partner violence, and age-disparate sex.

**Results:**

The final sample included 18 296 adolescent girls and young women (median [IQR] age, 19 [15-21] years) in 10 642 households. Approximately half of participants (49.9%; n = 8414) reported engaging in sexual activity, and 48.1% (n = 3946) reported condom use at the most recent sexual encounter. KwaZulu-Natal province had a higher HIV prevalence than Gauteng province (15.1% vs 7.8%; *P* < .001). Approximately one-fifth of participants (17.6%; n = 3291) were not exposed to any interventions, whereas 43.7% (n = 8144) were exposed to 3 or more interventions. There was no association between exposure to DREAMS-like interventions and HIV status. Adolescent girls and young women who accessed 3 or more interventions were more likely to have undergone HIV testing (adjusted odds ratio, 2.39; 95% CI, 2.11-2.71; *P* < .001) and to have used condoms consistently in the previous 12 months (adjusted odds ratio, 1.68; 95% CI, 1.33-2.12; *P* < .001) than those who were not exposed to any interventions.

**Conclusions and Relevance:**

Results of this study suggest that self-reported exposures to multiple or layered DREAMS-like interventions were associated with favorable behavioral outcomes. The beneficial aspects of layering HIV interventions warrant further research to support the sexual and reproductive health of adolescent girls and young women.

## Key Points

**Question:**

What is the association between exposure to multiple or layered interventions (such as the DREAMS [Determined, Resilient, Empowered, AIDS-free, Mentored, and Safe]-like program) and key biological and behavioral HIV-related outcomes?

**Findings:**

In this cross-sectional study of 18 296 individuals in 10 642 households in 2 provinces in South Africa, adolescent girls and young women who accessed 3 or more DREAMS-like interventions were significantly more likely to have undergone HIV testing and more likely to have used condoms consistently in the previous 12 months compared with those who did not attend such interventions.

**Meaning:**

Findings of this study suggest the need for further study of the beneficial aspects of layering HIV interventions to support the sexual and reproductive health of adolescent girls and young women.

## Teaser

This cross-sectional study assesses the use or availability of HIV-prevention initiatives, biological and behavioral risks, and HIV-related outcomes among female youth in 2 provinces in South Africa.

## Introduction

In South Africa, adolescent girls and young women aged 15 to 24 years are among the most high-risk population groups for acquiring HIV. In 2019, an estimated 70 000 adolescent girls and young women acquired HIV in South Africa,^1^ accounting for approximately 35% of all new HIV infections in the country. HIV prevalence among female individuals aged 15 to 19 years is currently 5.8% and peaks at 15.6% among those aged 20 to 24 years.^2^ The highest annual HIV incidence in any subpopulation is among adolescent girls and young women (1.5%) aged 15 to 24 years, with concomitant HIV-related deaths estimated at approximately 3900 per year.^2^

Numerous factors contribute to the high rates of HIV among adolescent girls and young women, including social (eg, gendered norms), behavioral (eg, sexual risk taking), and structural (eg, labor migration) factors.^3,4,5,6^ Furthermore, poor mental health has been associated with risk-taking behaviors that are linked to high rates of substance use, binge drinking, and sexual violence victimization.^7^ In this population, high rates of age-disparate sexual partnerships are associated with increased susceptibility to HIV acquisition^8,9,10,11^ because older men are more likely to have HIV-positive results and be unaware of their HIV status,^8,12^ and age-disparate partnerships are often characterized by inconsistent condom use^13,14^ and sequential or concurrent sexual partnering.^15^ In addition, adolescent girls and young women in South Africa are at an increased risk for experiencing intimate partner violence (IPV), which may heighten their risk of HIV infection.^16^ Many of these risk factors are compounded by cervical ectopy,^17^ which is a biological risk factor for acquiring HIV and other sexually transmitted infections (STIs).^18^

In response to the high and sustained HIV incidence among adolescent girls and young women in the southern and eastern African regions, and in South Africa particularly, the Determined, Resilient, Empowered, AIDS-free, Mentored, and Safe (DREAMS) program was established to empower and to reduce HIV incidence by 40% over a 2-year period (2016-2018) in this population.^19^ The DREAMS program aims to provide youth-friendly reproductive health care and social asset building; mobilize communities for change with school- and community-based HIV and violence prevention; reduce risk of sex partners through the US President's Emergency Plan for AIDS Relief program, including HIV testing, treatment, and voluntary medical male circumcision; and strengthen families with social protection (educational subsidies and combined socioeconomic approaches) and parental or caregiver programs.^19^ The layered (defined as multiple exposure), evidence-based HIV prevention interventions target a combination of biological, behavioral, and structural factors that address multiple HIV risk pathways simultaneously.^20^

The rollout of the DREAMS program in selected districts in South Africa (Gauteng and KwaZulu-Natal provinces) commenced on March 13, 2017. Data collection for the present study occurred between March 13, 2017, and June 22, 2018. In the study districts, we could not distinguish between DREAMS interventions and similar interventions that were implemented by the national government or other programs. Therefore, we use the term *DREAMS-like interventions* to be inclusive of all HIV prevention initiatives occurring within the study period.

In the present study, using a cross-sectional sample of adolescent girls and young women from selected districts in 2 South African provinces, we aimed to (1) describe HIV prevalence and HIV risk behaviors among a sample of adolescent girls and young women and (2) model the association between exposure to multiple or layered DREAMS-like interventions and key HIV biological and behavioral outcomes. The layered interventions included HIV testing; sexual reproductive health services; social-asset building; condom promotion and provision of preexposure prophylaxis; cash transfers and educational subsidies; parental or caregiver interventions; school-based HIV prevention; community-based HIV and violence prevention; social and gender norms change interventions; and interventions targeting partners with HIV prevention and care treatments, such as voluntary medical male circumcision and antiretroviral therapy.^21^

## Methods

### Study Design and Participants

This cross-sectional study was conducted from March 13, 2017, to June 22, 2018, in the Districts of Ekurhuleni and City of Johannesburg in Gauteng province and the Districts of uMgungundlovu and eThekwini in the KwaZulu-Natal province. Details about the survey design are provided in the study protocol.^22^ The study protocol, informed consent, and data collection forms were reviewed and approved by the University of KwaZulu-Natal Biomedical Research Ethics Committee, the US Centers for Disease Control and Prevention, and provincial health authorities in South Africa. All participants provided written informed consent. We followed the Strengthening the Reporting of Observational Studies in Epidemiology (STROBE) reporting guideline.

The 2 districts of interest within the KwaZulu-Natal province are among those with the highest HIV prevalence in South Africa: 20% in 2016 in uMgungundlovu and 16.7% in 2017 in eThekwini.^22^ Gauteng province has the fifth highest provincial HIV prevalence in the country (17.6% in 2017).^2^ The sample size calculations have been published elsewhere.^22^ Briefly, we used a stratified cluster random sampling design to target 18 500 adolescent girls and young women. The sample size per district was designed to be proportional to the estimated number of adolescent girls and young women in the DREAMS program subdistricts. Only small area layers in which the DREAMS program was implemented were included in the sampling frame. Small area layers were selected in proportion to the size of the sample required in each province to calculate HIV estimates; therefore, the Gauteng province, with an expected lower HIV incidence and prevalence, required a greater number of small area layers than did the KwaZulu-Natal province. All eligible adolescent girls and young women in a sampled household were invited to participate in the study. One parent or caregiver was interviewed in the household.

To be eligible for inclusion in this study, adolescent girls and young women had to be aged 12 to 24 years, willing to participate, legally able to provide written informed consent on their own or through their parent or caregiver, and willing to provide biological samples. For those younger than 18 years, parent or caregiver consent was obtained along with child assent.

### Measures

The independent variables were the multisector DREAMS-like interventions (eAppendix in the Supplement) consisting of a parent or caregiver intervention exposure index variable, which was created from the 20-item survey, that asked participants or parents or caregivers if they had attended certain interventions in the previous 12 months (eTable in the Supplement provides more information on each exposure variable). The DREAMS-like interventions were labeled and coded according to the DREAMS package category that was identified by Gourlay et al^21^ in their analysis of South African and Kenyan data. Participants were assigned a score of 1 for each DREAMS-like intervention to which they were exposed. Scores were aggregated and then used to create an index of intervention exposure: no exposure (0), 1-intervention exposure (1), 2-interventions exposure (2), and 3 or more–interventions exposure (3). This index assessed the association of layered interventions with outcome variables.

The primary outcome was HIV prevalence, which was ascertained from laboratory-based testing of peripheral blood samples. Secondary outcomes included HIV testing and antiretroviral therapy uptake, which were obtained by asking participants if they had tested for HIV in the previous 12 months and by quantitative testing for antiretroviral drugs of the blood samples of individuals with HIV-positive results, respectively. Other secondary HIV risk-related outcome variables included pregnancy, STI, IPV, age-disparate sex, number of sexual partners, and condom use in previous 12 months. Pregnancy was assessed by asking participants if they had ever been pregnant. For STIs, participants were asked if they had been diagnosed with an STI in the previous 12 months. For incidence of sexual and physical IPV, if the participants indicated experiencing 1 of the 13 IPV measures, they were coded as having experienced IPV.^16^ Age-disparate sex was assessed by asking if any of the participants’ sexual partners in the previous 12 months was 5 or more years older than them. We also asked about the number of sexual partners in their lifetime and condom use in the previous 12 months. HIV prevention knowledge was measured using a 6-item index focusing on abstinence, faithfulness to a single partner, condom use, whether a healthy person can have HIV, whether sleeping with a virgin can cure HIV, and whether witchcraft can cure HIV. We used the median score to create a cutoff for the HIV knowledge variable.

Secondary variables were the sociodemographic characteristics, including age, province of residence, home language, race and ethnicity (which were self-reported by participants and included the following categories: African, Asian or Indian, White, mixed racial and ethnic identity, and other [Zimbabwean, Mozambican, Malawian, Basotho, and Zulu]), educational level, whether they were currently repeating a grade, relationship status, and whether they had been away from home for more than 1 month in the previous year. Household-level information was also included in the parent or caregiver survey (which was separate from the survey for adolescent girls and young women), such as household income per month, whether the household received social grants (which are cash payments provided by the South African government directly to recipients; there are 7 types of social grants), and frequency of no food in the house in the previous 4 weeks. Several sexual behaviors were also analyzed, including age at first sexual encounter, engaging in transactional sex (defined as exchange of cash or goods for sex) in the previous 12 months, and contraceptive use.

### Statistical Analysis

Data analysis was conducted from March 12, 2021, to March 1, 2022, using SPSS, version 26 (IBM SPSS Statistics). SPSS complex sample procedures were used to account for multilevel sampling and study weights used. The sample weights facilitated the interpretation of results at the provincial level. The final sampling weight was the product of the small area layers’ weight, household weight, adjusted for individual nonresponse. The final individual weights were benchmarked to the 2018 Statistics South Africa midyear population estimates of adolescent girls and young women by age and province. Descriptive statistics with unweighted counts and population-weighted percentages with 95% CIs were calculated first. Then, multiple logistic regression analyses were undertaken to assess the association between DREAMS-like interventions uptake and HIV outcomes, while adjusting for several control variables.

Taylor series linearization methods were used to estimate SEs. We ascertained adjusted odds ratios (AORs) with 95% CIs for the multiple logistic regressions. Unpaired, 2-tailed *t* tests were used to calculate *P* values. A 2-sided *P* < .05 was considered statistically significant.

## Results

The final sample included 18 296 adolescent girls and young women with a median (IQR) age of 19 (15-21) years (eFigure in the Supplement). The survey was administered to 18 296 individuals in 10 642 households. Overall, 10 384 participants were enrolled in the Gauteng province and 7912 in the KwaZulu-Natal province. Table 1 provides the household data and individual sociodemographic characteristics, sexual risk behaviors, HIV knowledge index, and biological and clinical characteristics stratified by province.

**Table 1.** Household- and Individual-Level Characteristics Stratified by Province, 2017 to 2018

Characteristic	No. (%)		
	Gauteng province	KwaZulu-Natal province	Total
**Household data (N = 10 642)**			
Wealth status in previous 12 mo			
Became poorer	849 (8.2)	611 (8.4)	1460 (8.3)
Stayed the same	8722 (85.4)	6483 (83.4)	15 205 (84.7)
Became wealthier	669 (6.4)	628 (8.2)	1297 (7.0)
Receipt of social grants			
Yes	3154 (58.1)	2628 (71.8)	5782 (62.1)
No	2209 (39.8)	1001 (27.0)	3210 (36.1)
Do not know	89 (1.6)	29 (0.8)	118 (1.4)
Refused to answer	29 (0.5)	12 (0.3)	41 (0.5)
Household income per mo, R (US)^a^			
<1000 ($65)	1203 (19.6)	847 (18.3)	2050 (19.1)
1001-5000 ($65-$326)	3014 (50.2)	2620 (57.5)	5634 (52.7)
>5000 (>$326)	1002 (16.5)	772 (16.4)	1774 (16.5)
Do not know	727 (12.0)	293 (6.6)	1020 (10.2)
Refused to answer	107 (1.8)	57 (1.2)	164 (1.6)
Frequency of no food in household in previous 4 wk			
Often	429 (6.9)	214 (4.7)	643 (6.2)
Sometimes	879 (14.4)	533 (11.3)	1412 (13.4)
Rarely	560 (9.4)	426 (10.1)	986 (9.7)
Never	4185 (69.2)	3416 (73.9)	7601 (70.8)
**Individual participant data (N = 18 296)**			
Sociodemographic characteristic			
Age,y			
Median (IQR)	19 (15-22)	19 (15-21)	19 (15-21)
12-14	2350 (18.6)	1820 (19.4)	4170 (18.9)
15-19	3992 (36.2)	3006 (38.0)	6998 (36.8)
20-24	4042 (45.2)	3086 (42.6)	7128 (44.3)
Home language			
Zulu	5187 (49.7)	7675 (96.8)	12 862 (65.8)
Xhosa	810 (7.7)	134 (1.7)	944 (5.6)
Sotho	1863 (18.1)	56 (0.8)	1919 (12.2)
English	202 (2.2)	37 (0.6)	239 (1.6)
Afrikaans	224 (2.2)	1 (0)	224 (1.5)
Tswana	1049 (10.2)	9 (0.1)	1050 (6.7)
Other^b^	1049 (10.0)	0	1058 (6.6)
Race and ethnicity^c^			
African	10 052 (96.6)	7879 (99.5)	17 931 (97.6)
Asian or Indian	4 (0)	18 (0.3)	22 (0.1)
White	3 (0)	1 (0)	4 (0)
Mixed racial and ethnic identity	317 (3.3)	12 (0.2)	329 (2.2)
Other^c^	8 (0.1)	2 (0)	10 (0.1)
Educational level			
No or preprimary schooling	347 (3.8)	220 (2.9)	567 (3.5)
Did not complete formal education	5638 (50.8)	4371 (55.3)	10 009 (52.3)
Completed formal education	2852 (30.0)	2181 (29.0)	5033 (29.7)
Did not complete or completed tertiary education	1278 (13.9)	839 (11.1)	2117 (13.0)
Other^d^	144 (1.6)	85 (1.6)	229 (1.6)
Currently repeating a grade			
No	5665 (89.5)	4427 (91.8)	10 092 (90.3)
Yes	632 (10.5)	367 (8.2)	999 (9.7)
Away from home >1 mo in previous 12 mo			
No	9752 (93.5)	7465 (93.7)	17217 (93.6)
Yes	617 (6.4)	437 (6.2)	1054 (6.3)
Refused to answer	15 (0.1)	10 (0.1)	25 (0.1)
Current relationship status^e^			
Single	4984 (44.8)	3891 (46.3)	8875 (45.3)
Dating but not cohabiting^f^	4754 (48.3)	3858 (51.3)	8612 (49.4)
Dating and cohabiting^f^	510 (5.4)	97 (1.4)	607 (4.0)
Sexual risk behaviors			
Has had sex	4789 (50.3)	3625 (49.1)	8414 (49.9)
Aged <15 y at first sexual encounter	824 (16.2)	555 (16.5)	1379 (16.3)
Sexual partner ≥5 y older	1627 (34.5)	971 (27.8)	2598 (32.3)
Used condom at most recent sexual encounter	2397 (49.9)	1549 (44.5)	3946 (48.1)
Consistent condom use in previous 12 mo	1131 (23.5)	504 (14.5)	1635 (20.6)
IPV incidence			
Did not experience physical or sexual IPV	3839 (81.5)	3059 (87.6)	6898 (83.5)
Experienced physical or sexual IPV	853 (18.5)	410 (12.4)	1263 (16.5)
HIV knowledge index			
Poor^g^	5132 (47.6)	3240 (41.0)	8372 (45.3)
Moderate to good^g^	5254 (52.4)	4651 (59.0)	9905 (54.7)
Biological and clinical characteristics			
Self-reported HIV testing status			
Not tested	1398 (17.7)	1028 (20.8)	2426 (18.7)
Tested	5520 (79.9)	3542 (76.3)	9062 (78.8)
Refused to reveal status	179 (2.3)	137 (3.0)	316 (2.5)
Laboratory-derived HIV status^h^			
HIV-negative test result	9598 (92.2)	6753 (85.9)	16 351 (89.7)
HIV-positive test result	761 (7.8)	1131 (15.1)	1892 (10.3)
HIV incidence	31 (0.86)	21 (0.91)	52 (0.87)
Current HIV treatment			
Not using ART	404 (53.3)	455 (39.6)	859 (46.4)
Using ART	358 (46.7)	675 (53.6)	1033 (53.6)
Pregnancy history			
Been pregnant	2348 (51.7)	2186 (64.2)	4534 (55.8)
Never been pregnant	2315 (48.3)	1268 (35.8)	3583 (44.2)
Self-reported STI			
With STI	387 (8.3)	300 (9.5)	687 (8.7)
Without STI	4276 (91.7)	2991 (90.5)	7267 (91.3)
Contraception, any type			
Using contraceptives	2944 (30.8)	2214 (30.4)	5158 (30.7)
Not using contraceptives	7440 (69.2)	5698 (69.6)	13 138 (69.3)

Abbreviations: ART, antiretroviral therapy; IPV, intimate partner violence; R, South African rand; STI, sexually transmitted infection.

^a^ The exchange rate at the time of this study was R15.36 to US $1.

^b^ Other languages included Venda, Chewa, Ndebele, Northern Sotho, Tsonga, Ndau, Portuguese, Shona, Swazi.

^c^ Race and ethnicity were self-reported by participants. Other category included people who listed their race and ethnicity as Zimbabwean (n = 2), Mozambican (n = 3), Malawian (n = 1), Basotho (n = 1), and Zulu (n = 2).

^d^ Other educational attainment included currently undertaking Adult Basic Education and Training and currently undertaking the National Accredited Technical Education Diploma.

^e^ Does not add up to 100% because some categories were excluded.

^f^ Dating was defined as engaging in a casual or serious romantic relationship.

^g^ HIV knowledge was categorized as poor if participants scored 0 to 3 out of 6 items on the HIV knowledge index, and as moderate or good if they scored 4 to 6.

^h^ Fifty-three individuals had no laboratory-derived HIV status data and thus were excluded from the calculations.

More than half of all households (52.7%; n = 5634) earned R1001 to R5000 South African rand (US $65-$326) per month. Most households (70.8%; n = 7601) reported never running out of food in the previous 4 weeks. Almost half of the participants were aged 20 to 24 years (44.3%; n = 7128) and were dating but not cohabiting with their partner (49.4%; n = 8612). Half of the participants (49.9%; n = 8414) had previously engaged in sexual activity, and nearly half (48.1%; n = 3946) reported using a condom at their most recent sexual encounter. Less than a quarter of sexually active participants (20.6%; n = 1635) used condoms consistently in the previous 12 months. Nearly one-third of participants (32.3%; n = 2598) reported engaging in age-disparate sexual relationships.

**Figure 1.**
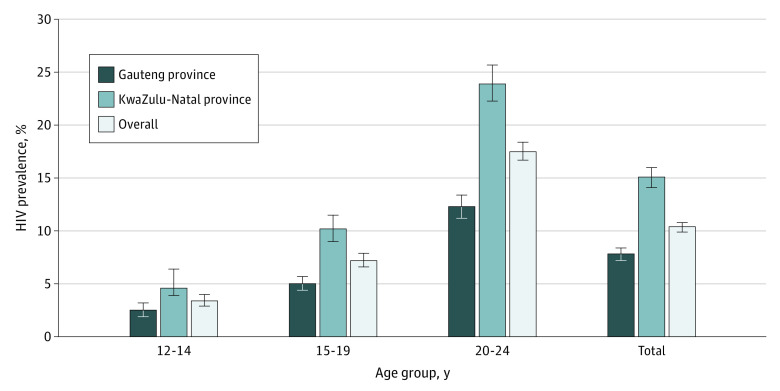
Weighted HIV Prevalence by Age and Province, 2017 to 2018 Error bars indicate 95% CIs.

The KwaZulu-Natal province had a higher HIV prevalence than Gauteng province (15.1% vs 7.8%; *P* < .001). The highest HIV prevalence for any age category was among those aged 20 to 24 years (23.9% in the KwaZulu-Natal province and 12.3% in the Gauteng province) (Figure 1).

Table 2 highlights the exposure of adolescent girls and young women to DREAMS-like interventions. The highest-reported exposure was to the school-based HIV intervention, with 63.2% of the participants (n = 11 495) being exposed in the previous 12 months. The second highest-reported exposure was to HIV testing, with 51.5% participants (n = 9516) attending educational interventions. Parental or caregiver interventions (0.8%; n = 159) and postviolence care (1.2%; n = 267) had the lowest exposure among participants and their parents or caregivers. Less than one-fifth of these respondents (17.6%; n = 3291) were not exposed to any interventions, whereas 43.7% of the participants (n = 8144) reported exposure to 3 or more interventions.

**Table 2.** Exposure to Interventions by Age Group, 2017 to 2018

DREAMS-like intervention	Age group							
	12-14 y		15-19 y		20-24 y		Total	
	No.	% (95% CI)	No.	% (95% CI)	No.	% (95% CI)	No.	% (95% CI)
HIV testing exposure	2033	48.3 (46.3-50.2)	3821	54.3 (52.7-56.0)	3662	50.6 (49.0-52.2)	9516	51.5 (50.3-52.8)
Social-asset building exposure	1059	25.5 (23.9-27.2)	1762	25.0 (23.6-26.4)	1573	22.0 (20.6-23.3)	4394	23.8 (22.7-24.8)
Expanded contraceptives mix exposure	1092	25.5 (23.8-27.2)	2587	36.4 (34.9-38.0)	2860	39.2 (37.6-40.8)	6539	35.6 (34.4-36.8)
Condom promotion or provision exposure	1982	47.3 (45.3-49.2)	3633	51.6 (50.0-53.1)	2766	37.5 (36.0-39.1)	8381	44.5 (43.3-45.7)
PrEP provision exposure	76	2.1 (1.5-2.6)	330	5.2 (4.6-5.8)	552	8.4 (7.6-9.2)	958	6.0 (5.5-6.5)
Social protection exposure	272	6.4 (5.5-7.2)	406	5.9 (5.3-6.5)	251	3.7 (3.2-4.3)	929	5.0 (4.6-5.5)
Postviolence care exposure	131	2.9 (2.3-3.6)	121	1.6 (1.2-2.1)	15	0.2 (0.0-0.3)	267	1.2 (1.0-1.5)
Parental or caregiver interventions exposure	77	2.0 (1.4-2.6)	77	1.1 (0.9-1.4)	5	0.1 (0.0-0.1)	159	0.8 (0.6-1.0)
School-based HIV prevention exposure	2603	62.7 (60.8-64.6)	4683	67.5 (66.0-69.0)	4209	59.9 (58.0-61.8)	11 495	63.2 (62.0-64.5)
Community mobilization exposure	141	3.6 (3.0-4.3)	129	1.9 (1.5-2.3)	19	0.3 (0.1-0.4)	289	1.5 (1.3-1.8)
Overall exposure to HIV interventions								
No exposure	833	19.9 (18.4-21.5)	1128	15.7 (14.6-16.8)	1330	18.2 (16.9-19.4)	3291	17.6 (16.7-18.5)
1-Intervention exposure	756	18.0 (16.7-19.4)	1267	18.4 (17.3-19.5)	1811	26.2 (24.8-27.6)	3834	21.8 (20.9-22.7)
2-Interventions exposure	776	19.2 (17.8-20.6)	1174	17.2 (16.2-18.3)	1077	15.9 (14.9-17.0)	3027	17.0 (16.3-17.7)
≥3-Interventions exposure	1805	43.0 (41.1-44.9)	3429	48.7 (47.1-50.4)	2910	39.7 (38.1-41.3)	8144	43.7 (42.4-44.9)

Abbreviations: DREAMS, Determined, Resilient, Empowered, AIDS-free, Mentored, and Safe; PrEP, preexposure prophylaxis.

We also found differences in exposure to interventions by age group (Table 2). More than two-thirds of adolescent girls and young women aged 15 to 19 years (67.5%; n = 4683) compared with 59.9% (n = 4209) of those aged 20 to 24 years, attended a school-based intervention in the previous 12 months. Overall, among participants who reported no exposure to any HIV interventions, those in the 12- to 14-year age group had the highest proportion (19.9%; n = 833) and those in the 15- to 19-year age group had the lowest proportion (15.7%; n = 1128). Nearly half of all participants aged 15 to 19 years (48.7%; n = 3429) were exposed to 3 or more interventions, whereas 39.7% (n = 2910) of those aged 20 to 24 years were exposed to 3 or more interventions.

Table 3 shows the HIV prevalence by exposures to different interventions. Overall, adolescent girls and young women who were exposed to condom promotion or provision had a slightly lower HIV prevalence than those who were not exposed (9.4% [n = 793 of 7563] vs 11.0% [n = 1099 of 8788]). Participants who were exposed to social protection interventions had a lower HIV prevalence than those who were not exposed (6.2% [n = 57 of 871] vs 10.5% [n = 1835 of 15 480]). Those who were exposed to postviolence care were less likely to have HIV infection than those who were not exposed (6.4% [n = 16 of 248] vs 10.3% [n = 1876 of 16 103]). Similarly, participants with exposure to community mobilization were less likely to have HIV infection than those with no exposure to such campaigns (4.0% [n = 12 of 277] vs 8.2% [n = 930 of 10 545]). Disaggregation by province revealed fairly similar patterns to those found for the total sample. However, there was a negligible difference in HIV prevalence in the Gauteng province between those who were exposed and those who were not exposed to postviolence care (5.5% [n = 6 of 122] vs 7.8% [n = 755 of 9474]). Unlike the finding for the combined sample, participants in Gauteng province who were exposed to parental or caregiver interventions had a lower HIV prevalence compared with those who were not exposed to such interventions (2.3% [n = 3 of 107] vs 6.8% [n = 439 of 6525]).

**Table 3.** HIV Prevalence by Interventions and Province, 2017 to 2018

DREAMS-like intervention	Gauteng province		KwaZulu-Natal province		Total	
	HIV prevalence, % (95% CI)	No./total No.	HIV prevalence, % (95% CI)	No./ total No.	HIV prevalence, % (95% CI)	No./total No.
Social-asset building						
No exposure	7.8 (7.1-8.4)	582/7312	15.2 (14.2-16.2)	867/5103	10.3 (9.7-10.9)	1449/12 415
Exposure	8.0 (6.9-9.2)	179/2284	14.6 (12.6-16.5)	264/1652	10.3 (9.3-11.4)	443/3936
HIV testing						
No exposure	8.0 (7.2-8.9)	405/4931	15.5 (14.2-16.8)	507/2909	10.3 (9.6-11.0)	912/7840
Exposure	7.6 (6.8-8.4)	356/4665	14.7 (13.4-16.0)	624/3846	10.3 (9.6-11.0)	980/8511
Expanded contraceptives mix						
No exposure	7.6 (6.9-8.3)	499/6486	14.3 (13.2-15.5)	651/4095	9.7 (9.1-10.4)	1150/10 581
Exposure	8.2 (7.3-9.2)	262/3110	16.0 (14.4-17.7)	480/2660	11.3 (10.4-12.2)	742/5770
Condom promotion or provision						
No exposure	8.6 (7.8-9.4)	480/5462	16.3 (15.1-17.6)	619/3326	11.0 (10.3-11.7)	1099/8788
Exposure	6.8 (6.0-7.6)	281/4134	13.7 (12.4-15.0)	512/3429	9.4 (8.7-10.2)	793/7563
PrEP provision						
No exposure	7.7 (7.1-8.3)	708/9082	14.9 (14.0-15.8)	1063/6436	10.2 (9.6-10.7)	1771/15 518
Exposure	9.5 (6.9-12.1)	53/514	16.7 (12.3-21.1)	68/319	12.0 (9.7-14.3)	121/833
Social protection						
No exposure	8.0 (7.4-8.6)	736/9053	15.3 (14.3-16.2)	1099/6427	10.5 (9.9-11.1)	1835/15 480
Exposure	4.5 (2.7-6.3)	25/543	10.2 (7.0-13.5)	32/328	6.2 (4.6-7.9)	57/871
Postviolence care						
No exposure	7.8 (7.2-8.4)	755/9474	15.2 (14.2-16.1)	1121/6629	10.3 (9.8-10.9)	1876/16 103
Exposure	5.5 (1.1-10.0)	6/122	7.7 (2.7-12.6)	10/126	6.4 (3.1-9.7)	16/248
Parental or caregiver interventions						
No exposure	6.8 (6.1-7.5)	439/6525	11.3 (10.3-12.4)	495/4146	8.2 (7.6-8.8)	934/10 671
Exposure	2.3 (0.0-5.0)	3/107	12.3 (1.4-23.2)	5/44	4.9 (1.3-8.6)	8/151
School-based HIV prevention						
No exposure	8.0 (7.1-9.0)	280/3369	17.3 (15.9-18.8)	517/2612	11.6 (10.7-12.5)	797/5981
Exposure	7.7 (6.9-8.4)	480/6226	13.5 (12.3-14.6)	614/4141	9.5 (8.9-10.2)	1094/10 367
Community mobilization						
No exposure	6.8 (6.1-7.5)	436/6432	11.4 (10.4-12.5)	494/4113	8.2 (7.6-8.8)	930/10 545
Exposure	3.3 (0.2-6.3)	6/200	6.1 (1.0-11.3)	6/77	4.0 (1.3-6.6)	12/277
Overall exposure to HIV interventions						
No exposure	8.2 (6.8-9.5)	141/1656	17.1 (15.0-19.2)	246/1241	11.5 (10.3-12.7)	387/2897
1-Intervention exposure	9.2 (7.9-10.4)	207/2156	16.2 (14.2-18.1)	230/1230	11.2 (10.1-12.3)	437/3386
2-Interventions exposure	6.6 (5.4-7.8)	114/1772	14.0 (11.8-16.2)	145/985	8.8 (7.7-9.8)	259/2757
≥3-Interventions exposure	7.4 (6.6-8.3)	299/4012	14.1 (12.8-15.5)	510/3299	9.9 (9.2-10.7)	809/7311

Abbreviations: DREAMS, Determined, Resilient, Empowered, AIDS-free, Mentored, and Safe; PrEP, preexposure prophylaxis.

**Figure 2.**
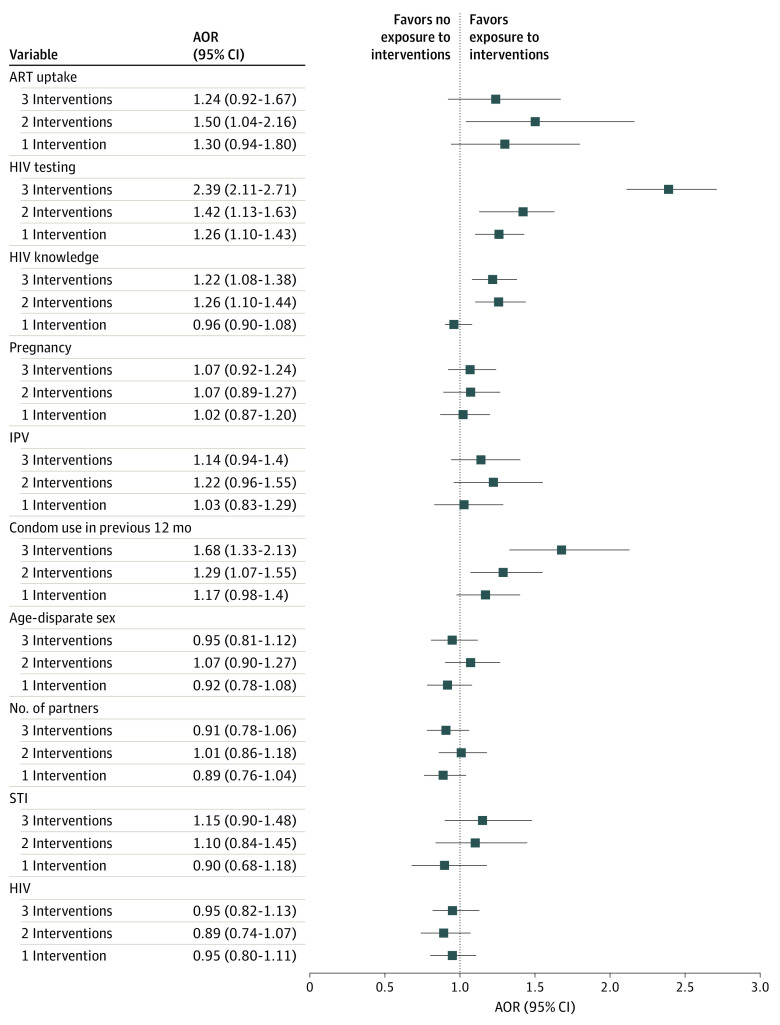
Forest Plot for the Weighted Logistic Regression for the Association of Interventions With HIV Status, HIV Risk, and HIV Care Outcomes, 2017 to 2018 Error bars indicate 95% CIs. AOR indicates adjusted odds ratio; ART, antiretroviral therapy; IPV, intimate partner violence; STI, sexually transmitted infection.

Figure 2 shows the associations of DREAMS-like interventions uptake with HIV status, HIV risk, and HIV care outcomes after adjusting for a number of covariates. There was no association found between DREAMS-like interventions uptake and HIV status. The logistic regression results revealed several associations between the exposure to DREAMS-like interventions and HIV testing, condom use, and HIV knowledge index. Adolescent girls and young women who accessed 3 or more interventions were more likely to have undergone HIV testing (AOR, 2.39; 95% CI, 2.11-2.71; *P* < .001), were more likely to have used condoms consistently in the previous 12 months (AOR, 1.68; 95% CI, 1.33-2.12; *P* < .001), and were more likely to have scored higher on the HIV knowledge index (AOR, 1.22; 95% CI, 1.08-1.38; *P* = .01), compared with those who were not exposed to any interventions. Individuals who attended 2 interventions were also more likely to have scored higher on the HIV knowledge index (AOR, 1.26; 95% CI, 1.10-1.44; *P* < .001) and to have been tested for HIV (AOR, 1.42; 95% CI, 1.13-1.63; *P* < .001) than those who were not exposed to any interventions. Those who reported exposure to 1 intervention (AOR, 1.26; 95% CI, 1.10-1.43; *P* = .003) were more likely to have undergone HIV testing than those without such exposure.

## Discussion

This cross-sectional study found a higher HIV prevalence in the KwaZulu-Natal province than in the Gauteng province. The highest HIV prevalence for any age category was among study participants aged 20 to 24 years. Exposures to some DREAMS-like interventions (eg, school-based HIV prevention [63.2%] and HIV testing [51.5%]) were high, whereas exposures to social protection (5.0%), parental or caregiver interventions (0.8%), and postviolence care (1.2%) were comparably low.

The results highlighted a lack of uniformity in the uptake of DREAMS-like interventions among adolescent girls and young women in these districts. The interventions with the greatest uptake were school-based HIV prevention, HIV testing, and expanded contraceptives. Such uptake may be attributed to individuals who were attending school-based sex education sessions and receiving contraceptives at the same time. We found low uptake of structural interventions, such as cash transfers, educational subsidies, and economic strengthening. Gaps in uptake of such key HIV risk-reduction interventions may partly explain the difficulties in reducing HIV infections among young women in South Africa over the past few decades.^23^ Although some local and national studies have shown a slight decrease in HIV incidence among adolescent girls and young women, progress toward reducing the rate of new HIV infections in this population has been slow.^2^

This study also found consistently low rates of condom use in the previous 12 months. Similar patterns were evidenced in the HIPSS (HIV Incidence Provincial Surveillance System) study.^23^ The more recent ECHO (Evidence for Contraceptive Options in HIV Outcomes) trial findings in South Africa also showed that, even when HIV prevention commodities such as condoms were available, condom use was low.^24^ Although there are many reasons for low or inconsistent condom use, adolescent girls and young women face a major challenge in convincing their male sexual partners to use condoms.^24^ Condom use campaigns should provide adolescent girls and young women with strategies for negotiating condom use with their partners. Another finding of this study was that a high proportion of participants (32.3%) were engaging in age-disparate sexual relationships. This pattern is concerning because previous research has suggested that adolescent girls and young women with male partners who are at least 5 years older than them are more likely to engage in condomless sex, transactional sex, more frequent sex, and/or concurrent sexual partnering.^15,25,26,27^

The results of this study indicated that layering (ie, exposure to multiple DREAMS-like interventions) was associated with a higher likelihood of undergoing HIV testing, accessing contraceptives in the previous 12 months, and attaining higher HIV knowledge index scores. Layering also appeared to have a greater effect size than exposure to only 1 intervention. Although the cross-sectional design precludes interpretations of causality, the findings of this study suggest that layered HIV interventions may be a useful strategy for supporting the sexual and reproductive health of adolescent girls and young women. This strategy has been confirmed by a study in Nairobi, Kenya, which found that the DREAMS program substantially increased knowledge of HIV status among adolescent girls and young women.^28^ More rigorous research is needed to identify the reason that layered interventions were not associated with the biological variables (eg, HIV status) and most behavioral variables (eg, age-disparate relationships and number of sexual partners), particularly longitudinal studies that address concerns about reverse causality. For example, we did not find any evidence of an association between exposure to DREAMS-like interventions and engaging in age-disparate relationships, but the reason may be that many adolescent girls and young women were already involved in age-disparate relationships before the DREAMS-like interventions were introduced. Furthermore, additional studies are warranted to assess the beneficial aspects of layering HIV interventions.

### Strengths and Limitations

The use of biomarker data is a strength of this study. This study also has several limitations. Previous research found a degree of underreporting on the sensitive behavioral, pregnancy, and STI data.^29^ The cross-sectional design of the present study means that it was not possible to draw causal inferences about the associations between variables. We selected participants from the DREAMS targeted areas; therefore, the results are not representative of the KwaZulu-Natal and the Gauteng provinces or the 2 districts in each province. Nevertheless, the results are representative of the areas in which the DREAMS program was implemented. The results for the intervention exposure items need to be interpreted with caution because the underreporting or overreporting of self-reported exposure to these interventions is unknown. It was not possible to isolate the DREAMS-like interventions from the HIV prevention interventions that were delivered through a different program.

## Conclusions

This cross-sectional study found high self-reported exposures to some DREAMS-like interventions (eg, school-based HIV prevention) among adolescent girls and young women in this survey. Meanwhile, exposures to social protection, parental or caregiver interventions, and postviolence care were comparably low. There was evidence of an association between layered interventions and favorable behavioral outcomes, but further research is needed to assess the beneficial aspects of layering HIV interventions to support the sexual and reproductive health of adolescent girls and young women.

## Notes

Supplement. **eAppendix.** DREAMS Intervention Information **eTable.** Classification of Exposure Variables **eFigure.** Sampling Strategy
