# Supplementary material for: Association of HIV Intervention Uptake With HIV Prevalence in Adolescent Girls and Young Women in South Africa
Source: JAMA Netw Open. 2022 Apr 22;5(4):e228640. doi: 10.1001/jamanetworkopen.2022.8640 (PMC9034400; doi:10.1001/jamanetworkopen.2022.8640)
Supplement: Supplement. — eAppendix. DREAMS Intervention Information eTable. Classification of Exposure Variables eFigure. Sampling Strategy [file jamanetwopen-e228640-s001.pdf]

Supplementary Online Content

Govender K, Beckett S, Reddy T, et al. Association of HIV intervention uptake with HIV prevalence in adolescent girls and young women in South Africa. *JAMA Netw Open*. 2022;5(4):e228640. doi:10.1001/jamanetworkopen.2022.8640

eAppendix. DREAMS Intervention Information

eTable. Classification of Exposure Variables

eFigure. Sampling Strategy

This supplementary material has been provided by the authors to give readers additional information about their work.

© 2022 Govender K et al. *JAMA Network Open.*

eAppendix. DREAMS Intervention Information[1]

DREAMS was launched by The United States President's Emergency Plan for AIDS Relief (PEPFAR) on the first of December 2014. The goal was to significantly reduce new HIV incidence among adolescent girls and young women in fifteen sub-Saharan Africa countries and in Haiti, where these girls face a higher risk for new HIV infection (2 to 14 times higher) than their male counterparts. DREAMS was created as a public-private partnership with the Bill & Melinda Gates Foundation, Gilead Sciences, Girl Effect, Johnson & Johnson, and ViiV Healthcare and PEPFAR. One billion United States Dollars has been invested into these DREAMS activities across the sixteen countries.

South Africa received 33 million United States Dollars for 5 years and in the sixth year they received 90 million United States Dollars (approximately 255 million United States Dollars in total)[1]. South Africa received the most funding from DREAMS out of all the countries. The province of KwaZulu-Natal has an overall prevalence of 15% amongst 15 to 49-year-old inhabitants in 2015. This is the highest prevalence in the country. The two districts of interest within KZN, uMgungundlovu (20% HIV prevalence in 2016) and eThekwini (16.8% HIV prevalence in 2016) are among those with the highest HIV prevalence in South Africa[2].The Gauteng province has the fifth highest HIV prevalence in the country with a prevalence of 11.1% in 2016. The HIV prevalence in the City of Johannesburg and Ekurhuleni is 11.1 and 14.3%, respectively[2].

As mentioned previously DREAMS seeks to layer multiple interventions for the young women. The layered approach includes behavioural, structural and biomedical interventions. The layered interventions include the provision of HIV testing services; sexual reproductive health services; social asset building; condom promotion and provision of pre-exposure prophylaxis (PReP); cash transfers and educational subsidies; parenting and caregiver interventions; school-based interventions; community-based HIV and violence prevention interventions; social and gender norms change interventions; and interventions targeting partners of AGYW with HIV prevention initiatives and HIV care interventions, such as voluntary medical male circumcision and antiretroviral therapy.

This approach is a strength of DREAMS but also very difficult to implement as implementers are not always used to following this approach. Therefore, the fidelity and quality of these interventions is not clear. DREAMS worked with the South African Department of Basic Education to ensure financial and programme sustainability. There were eleven different partners implementing different parts of the interventions. One of the weaknesses was passive referrals between community and clinical components in the DREAMS programme. DREAMS was implemented differently in the four districts and the same interventions were delivered differently by the 11 partners. This resulted in a lack of uniformity. One problem was meeting the need for DREAMS services in South Africa. Furthermore, there has been challenges in tracking the implementation of layered services. One positive outcome is a significant increase in the number of AGYW who have accessed PREP with 13 000 AGYW accessing PREP in 2019 compared to

[1] Most of the information for this section comes from the Fleischmann report which has been referenced.

© 2022 Govender K et al. *JAMA Network Open.*

only 500 in 2017[3]. Unfortunately, Covid-19 has had a negative impact on the DREAMS implementation, leading to a 49 percent decrease in completion of the DREAMS primary package[1].

eReferences

[1]Fleischman, J. 2021. Five Years of DREAMS and What Lies Ahead How to Address the Intersecting Crises of HIV, Gender Inequality, and Health Security. A report of the CSIS Global Health Policy Center. Available at: https://csis-website-prod.s3.amazonaws.com/s3fs-public/publication/210510_Fleischman_DREAMS_Five_Years.pdf?rbu98LOqCa5SPx0k60VJwIvvo7q.M5KP

[2] George, G., Cawood, C., Puren, A., Khanyile, D., Gerritsen, A., Govender, K., ... & Kharsany, A. (2020). Evaluating DREAMS HIV prevention interventions targeting adolescent girls and young women in high HIV prevalence districts in South Africa: protocol for a cross-sectional study. *BMC women's health*, *20*(1), 1-11.

[3] South Africa: DREAMS Overview (FY 2016–2021).2021. United States Department of State. Available at: https://www.state.gov/wpcontent/uploads/2020/06/SOUTH-AFRICA_DREAMSFact-Sheet-2020.pdf

© 2022 Govender K et al. *JAMA Network Open.*

eTable. Classification of Exposure Variables

DREAMS Package category	Variable in adolescent dataset	Variable in Caregiver dataset
HIV testing	HIV testing campaign	Received training/education on the benefit of HIV testing and getting to know your status?
Social asset building	Support group interventions for adolescents of same age meet.	Used-B-wise app;
Expand contraceptive mix	Contraception training/education	.
Condom promotion/provision	Condom training and education	.
Post violence care	.	Thuthuzela care centres
PREP	Ever used PREP	.
Social protection	Receive money to stay in school;	ASPIRES economic strengthening' programme; cash transfer or educational subsidy to help AGYW stay in school; any training related to parenting providing care to the children in your household?
Parenting/caregiver interventions	.	Family matters' programme; Project teens and adults learning to communicate' project
School-based HIV prevention interventions	Sexuality education;	Participated in 'Sexuality education in Life orientation' classes at school?
Community mobilisation	.	SASA Activist kit!, 'Vhutshilo^ 1' or 'Vhutshilo 2'; Stepping stones' interventions; Skillz intervention

Notes: classifications were taken from Gourlay et al. who conducted a similar study.

© 2022 Govender K et al. *JAMA Network Open.*

eFigure. Sampling Strategy

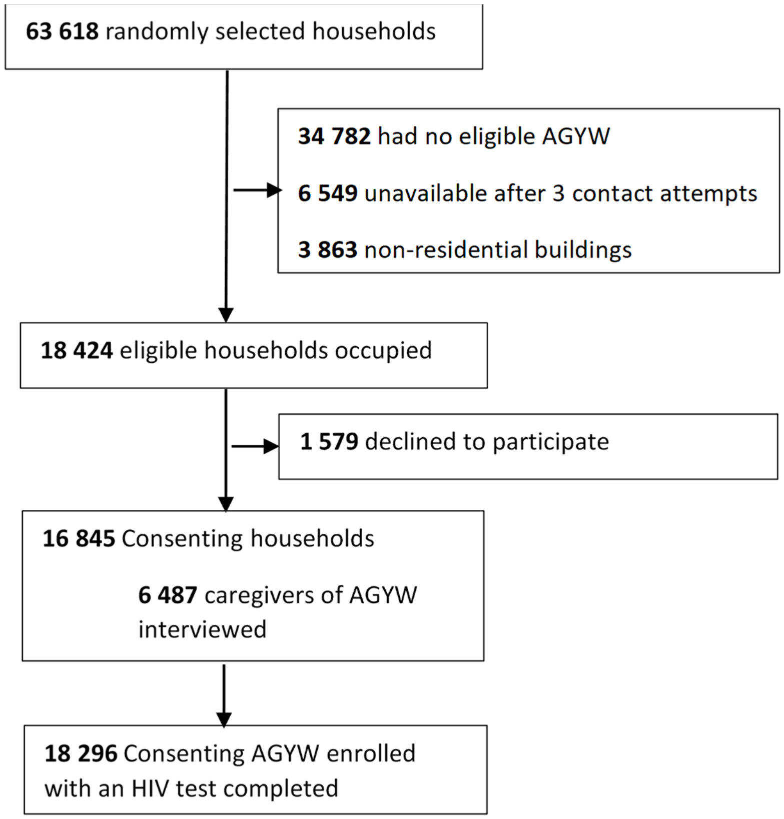

© 2022 Govender K et al. *JAMA Network Open.*
